# Action Video Gaming and Cognitive Control: Playing First Person Shooter Games Is Associated with Improved Action Cascading but Not Inhibition

**DOI:** 10.1371/journal.pone.0144364

**Published:** 2015-12-10

**Authors:** Laura Steenbergen, Roberta Sellaro, Ann-Kathrin Stock, Christian Beste, Lorenza S. Colzato

**Affiliations:** 1 Institute for Psychological Research, Leiden Institute for Brain and Cognition, Leiden University, Leiden, The Netherlands; 2 Cognitive Neurophysiology, Department of Child and Adolescent Psychiatry, Faculty of Medicine of the TU Dresden, Dresden, Germany; University of Ariel, ISRAEL

## Abstract

There is a constantly growing interest in developing efficient methods to enhance cognitive functioning and/or to ameliorate cognitive deficits. One particular line of research focuses on the possibly cognitive enhancing effects that action video game (AVG) playing may have on game players. Interestingly, AVGs, especially first person shooter games, require gamers to develop different action control strategies to rapidly react to fast moving visual and auditory stimuli, and to flexibly adapt their behaviour to the ever-changing context. This study investigated whether and to what extent experience with such videogames is associated with enhanced performance on cognitive control tasks that require similar abilities. Experienced action videogame-players (AVGPs) and individuals with little to no videogame experience (NVGPs) performed a stop-change paradigm that provides a relatively well-established diagnostic measure of action cascading and response inhibition. Replicating previous findings, AVGPs showed higher efficiency in response execution, but not improved response inhibition (i.e. inhibitory control), as compared to NVGPs. More importantly, compared to NVGPs, AVGPs showed enhanced action cascading processes when an interruption (stop) and a change towards an alternative response were required simultaneously, as well as when such a change had to occur after the completion of the stop process. Our findings suggest that playing AVGs is associated with enhanced action cascading and multi-component behaviour without affecting inhibitory control.

## Introduction

Cognitive control is defined as a set of processes that sustain our ability to interact with the environment in a goal-directed manner, by flexibly and continuously adapting our behaviour to the ever-changing environment [1]. As humans, we are regularly confronted with situations in which cognitive control is needed, for instance, when driving a car, cooking, doing sports, working, and in several other similar and more complex situations.

The importance of cognitive control processes becomes apparent when looking at the consequences its impairments can have on personal life and interpersonal relationships, as is the case for individuals with mental and neurological disorders (e.g., attention deficit/hyperactivity disorder (ADHD), obsessive–compulsive disorder, or dysexecutive syndrome [2–7]), in aging [8, 9], and for otherwise healthy individuals suffering from maladaptive habits (e.g., alcohol and substance abuse; [10–15]).

Given the important role of cognitive control processes in daily life, there is great interest in developing efficient methods to improve cognitive control functions and/or to counteract their decline. In this regard, action video game training seems to represent a promising tool [16]. Indeed, since the seminal work by Green and Bavelier [17], converging evidence has suggested that in contrast to other types of games, such as life-simulations, playing action video games (AVG)–in particular first-person shooter games such as the Halo, Call of Duty, and Battlefield series, and third-person shooter games such as the Gears of War and Grand Theft Auto series [18]–is associated with improvements in a wide range of perceptual [19–25], (visuo-)spatial [17, 26–30], perceptuo-motor [31–32] and attentional skills [17, 33–36]. For instance, AVG experience has been found to be associated with a more efficient distribution of visuo-spatial attention [17, 26], a general increase in central and peripheral visuospatial attention [27], an increment in the number of objects that can be apprehended [37], enhanced temporal processing of multisensory stimuli [20], enhanced sensorimotor learning [38], and a general speeding of perceptual reactions [39]. Remarkably, recent studies have complemented the aforementioned findings by showing that the beneficial effects of playing AVGs can generalize to cognitive control, that is, to people’s capacity to control their thoughts and action in a goal-directed manner. For instance, research has shown that AVG-players (AVGPs), compared to individuals with little to no videogame experience (NVGPs), have an enhanced ability to flexibly switch between tasks, as indexed by performance on a wide range of task-switching paradigms [40–48], which supports the idea that playing AVGs is associated with increased cognitive flexibility [43]. Moreover, AVGPs have been found to outperform NVGPs in the monitoring and updating of working memory (WM) representations [49]–another key cognitive-control function that is related to cognitive flexibility [50]. Conversely, inhibitory control (also considered an index of behavioural impulsivity) does not seem to be associated with AVGs experience. Indeed, a previous study [49] showed that playing AVGs results in more efficient response execution, but does not affect the ability to stop an ongoing response, as indexed by stop-signal reaction times (SSRTs; [51]) (for similar findings, see [52]). This latter finding is particularly intriguing. First, it questions the possibility that the beneficial effects of playing AVGs can transfer to all cognitive-control functions, as that would suggest AVGPs should also show superior inhibitory control (i.e., lower SSRTs) as compared to NVGPs. Second, it challenges the anecdotal idea that AVGPs are more impulsive than NVGPs, based on which AVGPs are expected to show lower inhibitory efficiency (i.e., higher SSRTs) than NVGPs.

In the present study we sought to complement previous findings by gaining a better understanding of the extent to which playing AVGs is associated with improved cognitive control. We focused on first person shooter (FPS) AVGs because it has been suggested that it is in particular the first person perspective that allows for cognitive-control improvements [43]. Indeed, compared to strategic and life-simulation games, the new generations of FPS AVGs are not just about pressing a button at the right moment, but they require the players to develop different action control strategies to rapidly react to fast moving visual and auditory stimuli, and to flexibly adapt their behaviour to the ever-changing context. This resembles complex daily life situations, such as multitasking conditions, in which we are required to inhibit a planned, ongoing response and to rapidly adapt our behaviour (e.g., to execute a different response). Successful performance under multitasking conditions relies on the ability to activate different task goals, and to cascade and prioritize different actions [53]. This leads to the possibility that extensive experience with playing FPS AVGs could be linked with better action cascading/multitasking performance. Yet, empirical evidence supporting this possibility is still missing.

Action cascading is defined as the ability to generate, process, and execute separate task goals and responses in an expedient temporal order and, as such, to be able to display efficient goal-directed multi-component behaviour [54–58]. The cascading of and selecting the right action can be done in a serial manner (i.e. step-by-step: a new task goal is activated only after the previous one has been carried out or stopped) or in a parallel manner (i.e. overlapping: a new task goal is activated while the previous one is still being is carried out), depending on the task demands [56–60].

**Fig 1:**
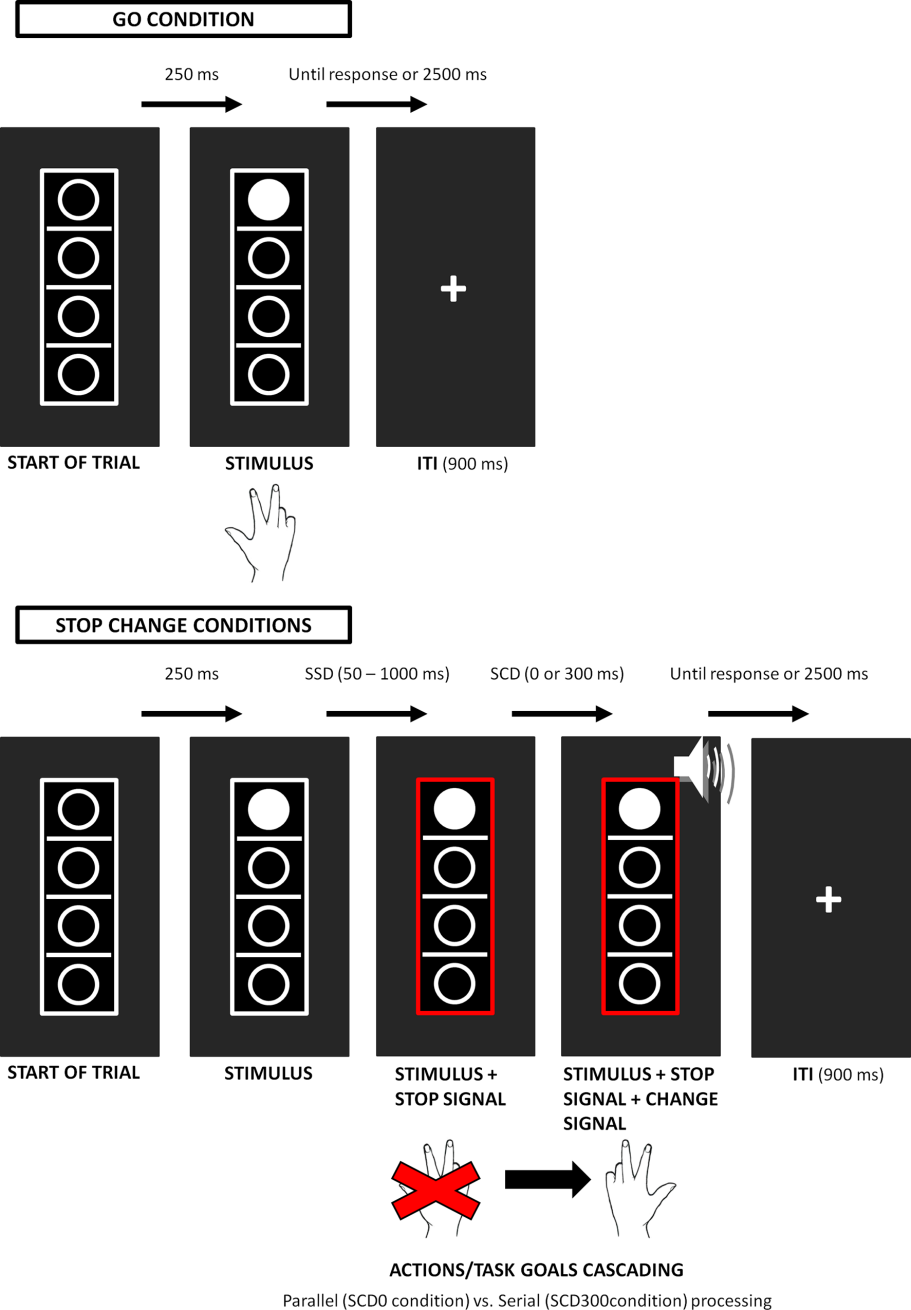
Schematic illustration of the stop-change paradigm. Circles indicate the four possible target locations, while the lines indicate the three possible reference lines. The red rectangle represents the STOP signal, the presentation of which (SSD) varied according to a staircase procedure (see text, for further details). The speaker icon represents the auditory CHANGE signal, which could be high (1300 Hz), medium (900 Hz) or low (500 Hz) in pitch. The pitch of the CHANGE signal indicates the new reference line to be used to judge the location (above vs. below) of the target stimulus (i.e., the white circle). The figure illustrates the sequence of the events (from left to right) for the GO condition (above panel) and for the STOP-CHANGE conditions (below panel). Each trial starts with the presentation of the four empty circles separated by three lines, with one of the circles becoming white after 250 ms. When no STOP signal is presented (i.e., GO condition–above panel), the presentation of the white circle (i.e., GO stimulus) requires participants to execute a right-handed response to judge its position with respect to the middle reference line. GO trials end after the response to the GO stimulus. Reaction times (RTs) on GO trials reflect the efficiency of response execution. When the STOP signal is presented (i.e., SC condition–below panel), participants are instructed to withdraw their right-handed response to the GO stimulus and to execute a left-handed response instead, judging the position of the white circle with respect to the new reference line (higher, middle, lower), as indicated by the pitch of the CHANGE signal (high, medium, low). The interval between the onset of the STOP and CHANGE stimuli (i.e., stop-change delay; SCD) was set to either 0 or 300 ms to create the SCD0 and SCD300 conditions. SC trials end after the response to the CHANGE stimulus. The time required to stop a planned/ongoing response (i.e., stop-signal reaction times, SSRTs) reflects inhibitory control efficiency. Responses on SC trials requires to inhibit a planned, ongoing response and to rapidly execute a different response. Successful performance on these trials relies on the ability to activate different task goals, and to cascade and prioritize different actions [53]. Therefore, RTs on these trials are indicative of the efficiency of action cascading, with shorter RTs indicating more efficient action cascading. ITI: intertrial interval; SSD: stop-signal delay; SCD: stop-change delay.

In order to assess whether extensive experience with AVGs can in fact result in an enhanced ability to prioritize and cascade different actions, we employed a stop-change task introduced by Verbruggen et al. [61]. In this task, the primary goal is to quickly react to a GO stimulus. Occasionally, a STOP stimulus is presented, which requires participants to stop the ongoing response. The STOP stimulus is followed by a CHANGE stimulus signalling the participants to shift to an alternative response. The interval between the STOP and the CHANGE stimulus (stop-change delay; SCD) hence, the duration of the preparation process before the execution of the change response, is manipulated in such a way that the two stimuli occur either simultaneously (0 ms; i.e., SCD 0) or with a short delay (300 ms; i.e., SCD 300; for more details, see Method section and Fig 1). Responses on SC trials depend on the ability to activate different task goals, and to cascade and prioritize different actions so as to succeed in inhibiting an ongoing response and rapidly switching to a different one [53]. As such, reaction times (RTs) on stop-change trials can be taken to reflect the efficiency of action cascading, with shorter RTs reflecting more efficient action selection.

Based on the available findings [40–46] we expected AVGPs to outperform NVGPs in action cascading processes (i.e., to show faster RTs on the stop-change trials) both when an interruption (stopping) and a change toward an alternative response are required simultaneously (SCD0) and when the change to another response is required when the stopping process has already finished (SCD300). Aside from providing a measure of action cascading efficiency, the stop-change paradigm also allows an assessment of the efficiency of response execution, as reflected by RTs to the GO stimuli, and a quantitative estimation of the duration of the covert response-inhibition process (i.e., the efficiency of inhibitory control), as indexed by the SSRTs (i.e., the time required to stop the ongoing response; [62–63]). Assuming we would replicate previous findings ([49], see also [50]), we expected AVGPs, compared to NVGPs, to show higher efficiency in response execution (i.e., faster RTs to the GO stimuli), but comparable performance on response inhibition (i.e., comparable mean SSRTs).

Finally, to rule out between-groups differences in terms of fluid intelligence, which could partially account for possible differences in cognitive control [64–65], we also assessed participant’s fluid intelligence by means of the Raven’s standard progressive matrices [66]. Building on previous studies [41, 43, 49, 67], AVGPs are expected to show comparable performance to that of NVGPs.

## Materials and Methods

### Participants

Thirty-six young healthy adults (28 men and 8 women) participated in the experiment. They constituted the two groups of 18 FPS AVGPs and 18 NVGPs. Participants were selected from a sample of 90 young adults who had previously participated in other studies in our lab and agreed to be contacted to participate in other behavioural studies. Using a covert recruitment strategy, the 90 potential volunteers were required (via e-mail) to fill in a questionnaire that assessed their experience with videogame along with other preferences (i.e., religious belief and preferred temperature). Specifically, participants were asked the following questions: (1) Are you baptized? (2) How often do you pray? (3) How often are you going to the church? (4) Do you prefer the heater high or low? (5) Do you work/study better when the heater is high or low? (6) Do you play video games? (7) Which kind of video games do you play and how much time do you spend playing them per week? (8) When did you start playing video games?. Following previous studies [17, 26, 27, 37], participants who reported playing a minimum of 5h/week of FPS AVGs, over the last year were defined as AVGPs. Twenty-two participants felt in this category and were invited to the lab, but only 18 of them showed up for the testing session. Participants assigned to the AVGP group reported to play FPS games such as Call of Duty, Unreal Tournament, Half-Life 2, and Battlefield 2 and later versions. All of these games are situated in a 3D environment and require frequent updating between multiple tasks and stimuli. Eighteen matched participants who reported little to no AVG experience (i.e., one or fewer hours per week on average of action videogame play) were selected to form the NVGP group.

All participants who were invited to the lab were also screened individually by a phone interview by the same lab-assistant using the Mini International Neuropsychiatric Interview (MINI) [68]–a well-established brief diagnostic tool used in clinical and stress research that screens for several psychiatric disorders and drug use [68–70].

Prior to the testing session, all participants were informed that they were participating in a study on the effects of playing videogames on cognitive performance. Written informed consent was obtained from all participants. The protocol and remuneration arrangements of 6.50 Euros were approved by the institutional review board (Leiden University, Institute for Psychological Research). The methods were carried out in accordance with the approved guidelines.

### Procedure

All participants were tested individually. Participants started with the practice procedure of the stop-change paradigm, which took about 20 minutes. After completion of the practice, participants performed the task (25 minutes) and filled out the short version (i.e., 30 items) of the Raven’s SPM (Standard Progressive Matrices) [66, 71], a standard and widely-used test to measure fluid intelligence [66]. Each participant was given 10 minutes to perform the SPM test. Participants were allowed to take a short break (maximum of 5 minutes) between tasks.

### Stop-Change paradigm

The task was adapted from Steenbergen et al. [60, 72] and Yildiz, Wolf and Beste [73], see Fig 1.

The experiment was controlled by an Asus laptop running on an Intel Core i3-3217U processor, attached to a LG Flatron 776FM 16 inch monitor (refresh rate of 60 Hz). Stimulus presentation and data collection were controlled using Presentation software system (Neurobehavioral Systems, Inc., Berkeley, CA). Responses were executed via button-presses using the number row of a QWERTY computer keyboard. Throughout the task, the response buttons were marked with yellow stickers. All visual stimuli were presented in white on a black background.

Each trial started with the presentation of four vertically aligned unfilled circles (diameter 7 mm) and three horizontal reference lines (line thickness 1 mm, width 13 mm), embedded in a 55 x 16 mm rectangle presented in the centre of the screen. After 250 ms, one of the circle was filled white (GO stimulus). In the GO condition (67% of the trials), participants were to indicate the position (above vs. below) of the white circle relative to the middle reference line. Specifically, participants were instructed to press the “7” key (for below) and the “8” key (for above) with the index and middle finger of their right hand, respectively. Stimuli were shown until response, but not longer than 2500 ms. Instructions emphasized both accuracy and speed. When RTs were longer than 1000 ms, the word “Quicker” was presented above the rectangle until the participant responded.

In the SC conditions, which corresponded to the remaining 33% of the trials, the presentation of the white GO stimulus was followed by a STOP signal (a red rectangle replacing the previous white frame), signalling the participants to try to inhibit their right-handed response to the GO stimulus. The delay between the onset of the GO stimulus and the onset of the STOP signal (i.e., the stop signal delay, SSD) was initially set to 250 ms and then dynamically adjusted using a staircase procedure to yield a 50% probability of successfully inhibiting the GO response (see [60, 73–74]).

Specifically, after a completely correct SC trial (i.e. no response to GO stimulus, no response prior to the CHANGE stimulus in the SCD300 condition (explained below) and a correct left hand response to the CHANGE stimulus), the SSD of the next SC trial increased by 50 ms. Conversely, after an incorrect SC trial (if any of the above criteria were not met), the SSD of the next SC trial decreased by 50 ms. Additionally, the following restriction was applied to this procedure: the SSD values could not fall below a value of 50 ms and could not exceed a value of 1000 ms. Participants were not informed about the staircase procedure, and were instructed not to wait for the stop signal. Regardless of the stopping (inhibitory) performance, every stop signal was associated with one of three possible CHANGE stimuli. The CHANGE stimuli consisted of 100 ms sine tones presented through headphones at 75 dB sound pressure level and could be high (1300 Hz), medium (900 Hz) or low (500 Hz) in pitch. The presentation of the CHANGE stimulus signalled the participants to execute a left-handed response requiring them to judge the position (above vs. below) of the white circle relative to a new reference line, as indicated by the pitch of the tone. The presentation of the high tone indicated the highest of the three lines as the new reference, the medium tone indicated the middle line and the low tone indicated the lowest line (see Fig 1). The three tones occurred with equal frequency. Participants were instructed to press the “1” key for stimuli located above the newly assigned reference line, and the “2” key for stimuli located below the newly assigned reference line, using the middle and index fingers of the left hand, respectively. The delay between the presentation of the STOP signal and the presentation of the CHANGE stimulus (i.e., the stop change delay, SCD) was manipulated to vary as follows. In half of the SC trials, there was a SCD with a stimulus onset asynchrony (SOA) of 300 ms between the STOP and the CHANGE signals (SCD300 condition); in the other half of SC trials, the STOP and CHANGE stimuli were presented simultaneously (SOA of 0 ms, SCD0 condition). RTs for the stop-change trials were measured from the onset of the CHANGE stimulus. When RTs for the stop-change trials were longer than 2000 the English word “Quicker” was presented above the rectangle until the participant responded. During the inter-trial interval (ITI) a fixation cross was presented in the centre of the screen for 900 ms. Overall, the task comprised 864 experimental trials and lasted about 25 minutes.

### Statistical analysis

A Chi-square test was used to compare gender distribution over the two groups. Independent samples t-tests or non-parametric Mann-Whitney U tests (in case of a violation of the normality assumption) were used to compare the two groups with regard to fluid intelligence, age, and the number of hours spent per week playing different game genres including shooter, strategy, and other games (i.e., role-playing, puzzle and sports games). To assess the effect of AVG practice on action cascading, mean RTs were submitted to a repeated-measures ANOVA with condition (GO, SCD0, SCD300) as within-subjects factor and group (AVGPs vs. NVGPs) as between-subjects factor. Greenhouse—Geisser correction was applied when the sphericity assumption was violated. Tukey HSD post-hoc tests were performed to clarify mean differences in case of significant interactions. Given that for the stop-change trials, the percentage of errors is mainly determined by a staircase procedure and, thus, is artificially fixed at approximately 50% [61], we only analysed the percentages of errors for the GO trials. The non-parametric Mann-Whitney U test was preferred over the independent samples t-test because of a small violation to the normality assumption. To index response inhibition, individual SSRTs for stop-signal trials were calculated, as indicated by Verbruggen, Schneider and Logan [61]. SSRTs were analyzed by means of the Mann-Whitney U test, as this variable was shown not to be normally distributed. A significance level of *p*<0.05 was adopted for all statistical tests.

## Results

The data reported in this paper are available through https://osf.io/5gtu9/. Table 1 shows demographic information and the behavioural parameters for the stop-change paradigm separately for the AVGPs and NVGPs group. No significant between group differences were found for age, Z = -.511, *p* = .628, gender, χ2(1, N = 36) = .643, *p* = .423, or fluid intelligence (IQ), *t*(34) = -.470, *p* = .641. Significant group differences were observed when comparing the two groups with respect to the hours spent at playing shooter, Z = -5.429, *p* < .001, strategic, Z = -2.272, *p* < .05, and other videogames, Z = -3.001, *p* < .01. In all cases, AVGPs reported to have more experience than NVGPs (see Table 1).

**Table 1:** Demographic characteristics and behavioural parameters for the stop-change paradigm for AVGPs and NVGPs (Mean ± SEM).

*Variables*	*AVGPs*	*NVGPs*
N [M:F]	18 [15:3]	18 [13:5]
Age	21.2±0.6	22.4±1.1
Fluid Intelligence	114±3.0	116±2.7
Hours per week spent playing		
First person shooter games*	9.8±1.6	0.1±0.1
Strategic games*	4.3±1.5	0.4±0.2
Other games*	7.7±1.6	2.6±1.2
STOP-CHANGE PARADIGM		
Mean stop-signal RT (SSRT)	274±12	280±18
Mean RTs on GO trials*	551±30	623±30
Mean RTs SCD 0*	1012± 63	1185±63
Mean RTs SCD 300*	823±63	1018±63

Significant group difference

* p < 0.05.

AVGPs: action videogame players, NVGPs: non videogame players, RT: reaction time, SSRT: stop signal reaction time, SCD: stop-change delay

RT analysis showed a main effect of trial type (GO vs. SCD0 vs. SCD300), *F*(1.515,39.126) = 135.234, *p* < .001, η^2^_p_ = .799, MSE = 31200.879. Tukey HSD post-hoc tests showed that RTs were longer in the SCD0 condition (1098±44), as compared to the SCD300 (920±45) and the GO condition (587±21) (both *p* < .001). The latter conditions (i.e., SCD300 and GO) also differed significantly from each other, *p* < .001. Crucially, as expected, the main effect of group was significant as well, *F*(1,34) = 4.746, *p* = .036, η^2^_p_ = .122, MSE = 122863.44, indicating that RTs in general where faster in the AVGP group (795ms) as compared to the NVGP group (942ms). Remarkably, the two-way interaction involving group and trial type was not significant, *F*(1.515,39.126) = 2.124, *p* = .151. Therefore, consistent with our expectations, AVGPs outperform NVGPs on both response execution and action cascading. Interestingly, such an improvement in action cascading was observed both when the shift to the alternate response was required to occur simultaneously to a stopping process (i.e., SCD0 condition) and when the stopping process was already finished (SCD300 condition). No differences between groups were observed with regard to the percentage of errors on the GO trials, *p* = .226.

Finally, replicating a previous finding [49], the analysis of the SSRTs, did not reveal differences between the two groups: the distribution of SSRTs was the same across groups, Z = -.063, *p* = .96.

### Additional analyses

Recent evidence suggests that many of the cognitive enhancements associated with AVG experience can be seen as reflecting the fact that AVG experience allows gamers to learn more quickly and effectively how to perform new tasks, rather than reflecting immediate transfer effects on new tasks [16, 38, 75]. Therefore, one may argue that the better performance shown by AVGPs may be due to faster learning rather than to better response execution and action cascading performance. To rule out this possibility, trials were divided into three blocks of 288 trials each. We then re-ran the RTs analysis with the inclusion of the additional within-subjects factor “block”. ANOVA confirmed the main findings, revealing significant main effects of trial type, *F*(1.148, 39.017) = 134.805, *p* < .001, η^2^_p_ = .799, MSE = 94109.532, and, crucially, group, *F*(1,34) = 4.695, *p* = .037, η^2^_p_ = .121, MSE = 370540.019, but no significant interaction between the two factors, F (1,34) = 2.116, *p* = .128. Additionally, a significant main effect of block was found, *F*(1.573,53.470) = 7.803, *p* = .002, η^2^_p_ = .187, MSE = 17794.567. Tukey HSD post-hoc tests showed that RTs decreased with increasing task experience (i.e., RTs were 889ms, 884ms and 831ms in block 1, block 2 and block 3, respectively). Post-hoc analyses revealed no significant difference between block 1 and block 2 (*p* = .96), whereas significant differences were observed between bock 2 and block 3 (*p* < .005), and between block 1 and block 3 (*p* < .005). More importantly, the factor block interacted neither with trial type, nor with group, Fs ≤ 2.323, *ps* ≥0.72, with the latter finding ruling out an interpretation of the observed group differences in terms of learning-related differences (see [76] for similar findings suggesting that performance on the stop-change paradigm is not sensitive to learning effects).

## Discussion

Research has suggested that playing AVGs can lead to improvements in perceptual [19–25], (visuo-)spatial [17, 26–30], perceptuo-motor [31–32] and attentional abilities [17,33–36], and that such improvements can also extend to cognitive control functions such as cognitive flexibility [40–48], and WM updating [49], but not inhibitory control [49–50]. The present study aimed to extend previous findings by determining the potential effect of FPS AVG playing experience on action cascading, which encompasses cognitive control processes such as task goal manipulation and action selection in multitasking contexts [53, 61]. To this end, AVGPs and NVGPs were confronted with a stop-change paradigm–a well-established diagnostic index of action cascading efficiency [61]. Interestingly, besides providing an index of action cascading efficiency, performance on this task gives additional information on the efficiency of response execution and inhibitory control, thereby providing us with the opportunity to confirm (or disconfirm) previous observations [49–50] while simultaneously extending them. Results showed that AVGPs outperformed NVGPs in action cascading efficiency. Indeed, as compared to NVGPs, AVGPs were found to be faster in switching to an alternative response, regardless of whether this shift was required to occur simultaneously to a stopping process (i.e., SCD0 condition–i.e., parallel processing) or when the stopping process had already finished (SCD300 condition–i.e., serial processing). Therefore, the present findings provide support for the idea that FPS AVG playing experience is likely to be associated with a more efficient ability in selecting and applying different action control strategies depending on the task demands. To some extent, this finding is not surprising if one considers that playing FPS AVGs explicitly requires the players to be able to rapidly and flexibly adapt their behaviour to the ever-changing context such that, very often, planned actions need to be withheld and rapidly replaced by others–an ability that the current findings suggest can transfer to cognitive tasks tapping similar skills. Our observations fit with previous reports that have associated AVG practice with enhanced cognitive flexibility, as indexed by performance on a wide range of task-switching paradigms [40–48] and WM updating, as indexed by performance on the 2-back task [49].

Importantly, in the present study, we also replicated previous observations suggesting that AVG experience is associated with higher efficiency in response execution, but does not affect inhibitory control. Indeed, consistent with a previous study using the stop-signal task [49], AVGPs showed faster RTs to GO signals, but were comparable to NVGPs in terms of SSRTs. As mentioned in the Introduction, the converging observations that AVGPs show comparable performance to NVGPs with respect to inhibitory performance in different paradigms [49–50] has a twofold importance. On the one hand, the fact that AVGPs are not better than NVGPs in inhibitory control means that the potential beneficial effects associated with gaming experience do not transfer to all cognitive functions. It would be valuable for future studies to shed light on why this is the case. On the other hand, the fact that AVGPs are not worse than NVGPs in inhibitory control do not provide any empirical support for the claim, often seen in the media, that AVGPs are more impulsive, antisocial, or aggressive than non-gamers (but see [77]). Indeed, our findings, along with previous ones [49–50], show that AVGPs do not show any dysfunctional impulsivity or impairment in response inhibition, as compared to NVGPs.

The current study has some limitations that warrant discussion. First and foremost, we acknowledge that no causal relation can be drawn between the observed between-groups differences and FPS AVG playing experience. Indeed, our investigation was restricted to how a history of video game experience is associated with action cascading processes, rendering our study correlational in nature–a methodological shortcoming common to most studies reporting gaming effects (for an extensive discussion of this issue, see [19, 78]). Therefore, one cannot rule out that the differences we found in the stop-change task are actually due to innate differences between the groups, such as pre-existing neuro-developmental factors and/or a particular pre-gaming learning experience, rather than due to gaming exposure. For instance, individuals with a genetic predisposition associated with better executive control functions might be drawn to video games more strongly, meaning that an effect of experience might actually represent a form of self-selection [43]. Interestingly enough, this possibility is in line with other findings showing that cognitive skills are significant predictors of gaming performance in FPS AVGs [79–81]. Likewise, it seems that gender differences in cognitive skills may be causally linked with the decision to play specific genres of games rather than others, which may explain why males mostly prefer action, shooter, sports, and fighter games, whereas females typically prefer puzzle, card and educational games [79–81]. The fact that the two groups did not differ in terms of age, gender, and fluid intelligence allows us to at least exclude the potential confounding influence of these variables. Among these factors, age is probably of particular importance, as a previous study has shown that action cascading performance declines with increasing age [82]. Furthermore, as expected, we found no group differences in terms of fluid intelligence and inhibitory control, replicating previous studies that found no association between these factors and gaming experience [41, 43, 49, 67]. Nevertheless, our study remains correlational in nature and therefore it is crucial for future studies to examine the possible causal nature of our observed group differences. To this end, it is highly advisable to carry out training studies wherein action cascading performance of NVGPs is trained via AVG and thereafter compared with that of NVGPs trained in with control intervention. Perhaps better, longitudinal studies could assess whether and to what extent pre-existing differences between AVGPs and NVGPs may account for the reported effects on cognitive performance, thereby providing this field of research with higher external validity. Lastly, it would also be interesting to assess whether individual differences in action cascading performance can predict gaming performance, as shown in [79–81].

Second, the fact that participants were aware of participating in a study on the effects of playing videogames on cognitive performance–another main methodological shortcoming in this field of research [19, 78]–leads to the possibility that the between-groups differences in action cascading performance might have been driven by specific expectations and motivational factors. In other words, one may argue that AVGPs outperformed NVGPs to conform with the expectations wrought by their group membership and/or because they were more motivated to perform well. However, as argued elsewhere [83–84], for such expectancies-driven effects to occur, participants have to be aware of the specific hypotheses under investigation and of how such hypotheses would translate to the data. Furthermore, this criticism neglects that fact NVGPs may be likewise motivated to perform better than AVGPs. In any case, the fact that the two groups differed only in specific skills such as response execution and action cascading, but not in inhibitory control and fluid intelligence, undermines an interpretation of our results in these terms. Notably, the lack of any AVGP/NVGP difference in tasks tapping inhibitory control and fluid intelligence is consistent with results from a previous study in which a completely covert recruitment strategy was used [49]. Nevertheless, it would be informative for follow-up studies to replicate our findings in a context in which participants are totally blind to the nature of the study.

A third limitation of the present study is the small sample size, although comparable to that of other studies e.g., [20, 35–36, 43, 47, 75, 85], including mostly male participants. Therefore, more studies are needed in order to verify the reliability and repeatability of our findings in larger samples, possibly balanced for gender. However, it is important to note that the possibility to test samples balanced for gender is limited by the fact that males are much more likely than females to report playing AVGs (see [79–81, 86] and above for a possible explanation of why this is the case) and, consequently, there do not seem to be enough females with AVG expertise to allow for gender-balanced groups. This is why in previous cross-sectional groups studies recruitment was restricted to male participants [20, 35–36, 47, 75]. In the present study, we decided to include female participants as well, given that a previous study using the same task has revealed no gender-related differences in action cascading performance [82]. Importantly, even though it is difficult to say whether and to which degree our findings might generalize to female players, the imbalance with respect to gender cannot account for the observed group differences, as the two groups were matched for gender. Again, training studies would be preferable, as they would overcome this limitation as well.

Fourth, in the present study we restricted our hypotheses to AVGPs who played FPS games. This is because it has been suggested that it is in particular the first person perspective (as in the FPS games) that allows for cognitive-control improvements [43]. Indeed, success in FPS games requires high levels of action control and a flexible mindset to rapidly react to moving visual and sudden acoustic events, and to switch back and forth between different subtasks, both in a serial and in a parallel manner. However, a more systematic investigation is needed to verify this hypothesis. Specifically, for such a hypothesis to be supported follow-up studies may consider to compare action cascading performance between FPS AVGPs and AVGPs who mainly play third-person shooter games and/or other types of AVGs. Related to the this point, it is important to mention that our sample of AVGPs also had more experience with several other types of game genres including strategic, role-playing, puzzle and sports games. Although this is consistent with previous studies (e.g. [20], [41]), it is difficult to ascertain whether the better action cascading performance shown by our AVGPs is specifically due to playing specifically FPS AVGs, other games, or a combination of them. Due to such potential within-group variance, it is possible that our study was underpowered and, therefore, led to an underestimated, relatively small effect size that might seem of little clinical significance. Future studies should aim to decrease within-group variance as much as possible.

A final limitation pertains to the fact that our conclusion that playing AVG is associated with improved action cascading performance relies on participants’ performance on a single task. To obtain more reliable results, it would be ideal for future studies to confront AVGPs and NVGPs with different tasks that are reckoned to assess action cascading/multitasking performance, as validated, for instance, by confirmatory statistical analyses (e.g., confirmatory factor analysis and/or structural equation modelling analyses; see [50] for an example of application of these methods).

In sum, our findings are promising in suggesting that playing AVG can be associated with enhanced action cascading performance, i.e. more efficient goal-directed multi-component behaviour. As such, our findings may represent an important first step in stimulating further research to assess whether videogames can be used to optimize cognitive control. Importantly, given the importance of action control in daily activities and the known difficulties shown by older adults in response selection and action cascading processes [82, 87–89], our findings can have important practical implications for designing intervention/training studies aimed at overcoming or slowing down action control deficits associated with aging.

## References

[1] Botvinick MM, Braver TS, Barch DM, Carter CS, Cohen JD. Conflict monitoring and cognitive control. Psychol Rev. 2001;108: 624 11488380 10.1037/0033-295x.108.3.624

[2] Konrad K, Gauggel S, Manz A, Scholl M. Lack of inhibition: a motivational deficit in children with attention deficit/hyperactivity disorder and children with traumatic brain injury. Child Neuropsychol. 200;6: 286–296. 10.1076/chin.6.4.286.3145 11992192

[3] Konrad K, Gauggel S, Manz A, Scholl M. Inhibitory control in children with traumatic brain injury (TBI) and children with attention deficit/hyperactivity disorder (ADHD). Brain Inj. 2000;14: 859–875. 11076133 10.1080/026990500445691

[4] Gauggel S, Rieger M, Feghoff TA. Inhibition of ongoing responses in patients with Parkinson's disease. J Neurol Neurosurg Psychiatry 2004;75: 539–544. 15026491 10.1136/jnnp.2003.016469 PMC1739013

[5] Chamberlain SR, Fineberg NA, Blackwell AD, Robbins TW, Sahakian BJ. Motor inhibition and cognitive flexibility in obsessive-compulsive disorder and trichotillomania. Am J Psychiatry. 2006;163: 1282–1284. 16816237 10.1176/ajp.2006.163.7.1282

[6] Martinussen R, Hayden J, Hogg-Johnson S, Tannock R. A meta-analysis of working memory impairments in children with attention-deficit/hyperactivity disorder. J Am Acad Child Adolesc Psychiatry. 2005;44: 377–384. 15782085 10.1097/01.chi.0000153228.72591.73

[7] Nakao T, Nakagawa A, Nakatani E, Nabeyama M, Sanematsu H, Yoshiura T et al Working memory dysfunction in obsessive–compulsive disorder: a neuropsychological and functional MRI study. J Psychiatric Res. 2009;43: 784–791. 10.1016/j.jpsychires.2008.10.013 19081580

[8] Gazzaley A. Cognitive control deficits in our ageing population In: Stuss DT, Knight RT, editors. Principles of Frontal Love Function. Oxford: Oxford University Press; 2013.

[9] Levy RA. Aging-associated cognitive decline. Int Psychogeriatr. 1994:6; 63–68. 8054494

[10] Curtin JJ, Fairchild BA. Alcohol and cognitive control: implications for regulation of behavior during response conflict. J Abnorm Psychol. 2003;112: 424 12943021 10.1037/0021-843x.112.3.424

[11] Ambrose ML, Bowden SC, Whelan G. Working memory impairments in alcohol‐dependent participants without clinical amnesia. Alcohol Clin Exp Res. 2001;25: 185–191. 11236831

[12] McCann UD, Mertl M, Eligulashvili V, Ricaurte GA. Cognitive performance in (±) 3, 4-methylenedioxymethamphetamine (MDMA,“ecstasy”) users: a controlled study. Psychopharmacol. 1999;143; 417–425. 10.1007/s002130050967 10367560

[13] Li CSR, Luo X, Yan P, Bergquist K, Sinha R. Altered impulse control in alcohol dependence: neural measures of stop signal performance. Alcohol Clin Exp Res. 2009;33: 740–750. 10.1111/j.1530-0277.2008.00891.x 19170662 PMC2697053

[14] Fillmore MT, Rush CR. Impaired inhibitory control of behavior in chronic cocaine users. J Alcohol Drug Depend. 2002;66: 265–273. 10.1016/s0376-8716(01)00206-x 12062461

[15] Gouzoulis-Mayfrank E, Daumann J, Tuchtenhagen F, Pelz S, Becker S, Kunert HJ, et al Impaired cognitive performance in drug free users of recreational ecstasy (MDMA). J Neurol Neurosurg Psychiatry. 2000;68: 719–725. 10811694 10.1136/jnnp.68.6.719 PMC1736948

[16] Green CS, Bavelier D. Action video game training for cognitive enhancement. Curr Opin Behav Sci. 2015;4: 103–108.

[17] Green CS, Bavelier D. Action video game modifies visual selective attention. Nature. 2003;423: 534–537. 12774121 10.1038/nature01647

[18] King G, Krzywinska T. ScreenPlay: Cinema/videogames/interfacings London: Wallflower Press; 2002.

[19] Boot WR, Blakely DP, Simons DJ. Do action video games improve perception and cognition? Front Psychol. 2011;2: 226 10.3389/fpsyg.2011.00226 21949513 PMC3171788

[20] Donohue SE, Woldorff MG, Mitroff SR. Video game players show more precise multisensory temporal processing abilities. Atten Percept Psychophys. 2010;72: 1120–1129. 10.3758/APP.72.4.1120 20436205 PMC3314265

[21] Green CS, Pouget A, Bavelier D. Improved probabilistic inference as a general learning mechanism with action video games. Curr Biol. 2010;20: 1573–1579. 10.1016/j.cub.2010.07.040 20833324 PMC2956114

[22] Green CS, Li R, Bavelier D. Perceptual learning during action video game playing. Top Cogn Sci. 2010;2: 202–216. 10.1111/j.1756-8765.2009.01054.x 25163784

[23] Buckley D, Codina C, Bhardwaj P, Pascalis O. Action video game players and deaf observers have larger Goldmann visual fields. Vis Res. 2010;50: 548–556. 10.1016/j.visres.2009.11.018 19962395

[24] Appelbaum LG, Cain MS, Darling EF, Mitroff SR. Action video game playing is associated with improved visual sensitivity, but not alterations in visual sensory memory. Atten Percept Psychophys. 2013;75: 1161–1167. 10.3758/s13414-013-0472-7 23709062

[25] Li R, Polat U, Makous W, Bavelier D. Enhancing the contrast sensitivity function through action video game training. Nat Neurosci. 2009;12: 549–551. 10.1038/nn.2296 19330003 PMC2921999

[26] Green CS, Bavelier D. Effect of action video games on the spatial distribution of visuospatial attention. J Exp Psychol-Hum Percept Perform. 2006;32: 1465–1468. 17154785 10.1037/0096-1523.32.6.1465 PMC2896828

[27] Green CS, Bavelier D. Action-video-game experience alters the spatial resolution of attention. Psychol Sci. 2007;18: 88–94. 17362383 10.1111/j.1467-9280.2007.01853.x PMC2896830

[28] Spence I, Yu JJ, Feng J, Marshman J. Women match men when learning a spatial skill. J Exp Psychol Learn Mem Cogn. 2009;35: 1097 10.1037/a0015641 19586273

[29] Spence I, Feng J. Video games and spatial cognition. Rev Gen Psycho. 2010;14: 92.

[30] Feng J, Spence I, Pratt J. Playing an action video game reduces gender differences in spatial cognition. Psychol Sci. 2007;18: 850–855. 17894600 10.1111/j.1467-9280.2007.01990.x

[31] Hubert-Wallander B, Green CS, Sugarman M, Bavelier D. Changes in search rate but not in the dynamics of exogenous attention in action videogame players. Atten Percept Psychophys. 2011;73: 2399–2412. 10.3758/s13414-011-0194-7 21901575

[32] Chen R, Chen J, Li L. Action Videogame Play Improves Visual Motor Control. J Vis. 2015;15: 42 10.1167/15.12.42

[33] West GL, Stevens SA, Pun C, Pratt J. Visuospatial experience modulates attentional capture: Evidence from action video game players. J Vis. 2008;8: 13. 10.1167/8.16.13 19146279

[34] Hubert‐Wallander B, Green CS, Bavelier D. Stretching the limits of visual attention: the case of action video games. Wiley Interdiscip Rev Cogn Sci. 2011;2, 222–230. 10.1002/wcs.116 26302012

[35] Chisholm JD, Hickey C, Theeuwes J, Kingstone A. Reduced attentional capture in action video game players. Atten Percept Psychophys. 2010;72: 667–671. 10.3758/APP.72.3.667 20348573

[36] Chisholm JD, Kingstone A. Improved top-down control reduces oculomotor capture: The case of action video game players. Atten Percept Psychophys. 2012;74: 257–262. 10.3758/s13414-011-0253-0 22160821

[37] Green CS, Bavelier D. Enumeration versus multiple object tracking: The case of action video game players. Cognition. 2006;101: 217–245. 16359652 10.1016/j.cognition.2005.10.004 PMC2896820

[38] Gozli DG, Bavelier D, Pratt J. The effect of action video game playing on sensorimotor learning: evidence from a movement tracking task. Hum Mov Sci. 2014;38: 152–162. 25318081 10.1016/j.humov.2014.09.004

[39] Dye MW, Green CS, Bavelier D. Increasing speed of processing with action video games. Curr Dir Psychol Sci. 2009;18: 321–326. 20485453 10.1111/j.1467-8721.2009.01660.x PMC2871325

[40] Boot WR, Kramer AF, Simons DJ, Fabiani M, Gratton G. The effects of video game playing on attention, memory, and executive control. Acta Psychologica. 2008;129: 387–398. 10.1016/j.actpsy.2008.09.005 18929349

[41] Cain MS, Landau AN, Shimamura AP. Action video game experience reduces the cost of switching tasks. Attent PerceptPsychophys. 2012;74: 641–647. 10.3758/s13414-012-0284-1 22415446

[42] Colzato LS, van den Wildenberg W, Hommel B. Cognitive control and the COMT Val158Met polymorphism: Genetic modulation of videogame training and transfer to task-switching efficiency. Psychol. Res 2014;78: 670–678. 10.1007/s00426-013-0514-8 24030137

[43] Colzato LS, van Leeuwen PJA, van den Wildenberg WPM, Hommel B. DOOM’d to switch: Superior cognitive flexibility in players of first person shooter games. Front Psychol. 2010;1: 8 10.3389/fpsyg.2010.00008 21833191 PMC3153740

[44] Green CS, Sugarman M, Medford K, Klobusicky E, Bavelier D. The effect of action video game experience on task-switching. Comput Human Behav. 2012;28: 984–994. 22393270 10.1016/j.chb.2011.12.020 PMC3292256

[45] Karle JW, Watter S, Shedden JM. Task switching in video game players: Benefits of selective attention but not resistance to proactive interference. Acta Psychologica. 2010;134: 70–78. 10.1016/j.actpsy.2009.12.007 20064634

[46] Strobach T, Frensch PA, Schubert T. Video game practice optimizes executive control skills in dual-task and task switching situations. Acta Psychologica. 2012;140: 13–24. 10.1016/j.actpsy.2012.02.001 22426427

[47] Green CS, Sugarman MA, Medford K, Klobusicky E, Bavelier D. The effect of action video game experience on task-switching. Comput Human Behav. 2012; 28: 984–994. 22393270 10.1016/j.chb.2011.12.020 PMC3292256

[48] Andrews G, Murphy K. Does video-game playing improve executive function? In: Vanchevsky MA, editor. Frontiers in cognitive science. New York: Nova Science Publishers Inc; 2006 pp. 145–161.

[49] Colzato LS, van den Wildenberg W, Zmigrod S, Hommel B. Action video gaming and cognitive control: Playing first person shooter games is associated with improvement in working memory but not action inhibition. Psychol Res. 2013;77: 234–239. 10.1007/s00426-012-0415-2 22270615

[50] Miyake A, Friedman NP, Emerson MJ, Witzki AH, Howerter A, Wager TD. The unity and diversity of executive functions and their contributions to complex “frontal lobe” tasks: A latent variable analysis. J Cogn Psychol. 2000;41: 49–100. 10.1006/cogp.1999.0734 10945922

[51] Logan GD, Schachar RJ, Tannock R. Impulsivity and inhibitory control. Psychol Sci. 1997;8: 60–64.

[52] Dye MWG, Green SC, Bavelier D. Increasing speed of processing with action video games. Curr Dir Psychol Sci. 2009;18: 321–326. 20485453 10.1111/j.1467-8721.2009.01660.x PMC2871325

[53] Boecker M, Gauggel S, Drueke B. Stop or stop-change—Does it make any difference for the inhibition process?. Int J Psychophysiol. 2013;87: 234–243. 10.1016/j.ijpsycho.2012.09.009 23026439

[54] Dippel G, Beste C. A causal role of the right inferior frontal cortex in the strategies of multi-component behaviour. Nat Commun. 10.1038/ncomms7587 25850926

[55] Duncan J. The multiple-demand (MD) system of the primate brain: mental programs for intelligent behaviour. Trends Cogn Sci. 2010;14; 172–179. 10.1016/j.tics.2010.01.004 20171926

[56] Mückschel M, Stock A-K, Beste C. Psychophysiological mechanisms of interindividual differences in goal activation modes during action cascading. Cereb Cortex. 2014;24: 2120–2129. 10.1093/cercor/bht066 23492952

[57] Stock A-K, Blaszkewicz M, Beste C. Effects of binge drinking on action cascading processes: an EEG study. Arch Toxicol. 2014; 88: 475–488. 10.1007/s00204-013-1109-2 23925690

[58] Stock A-K, Arning L, Epplen JT, Beste C. DRD1 and DRD2 Genotypes Modulate Processing Modes of Goal Activation Processes during Action Cascading. J Neurosci. 2014;34: 5335–5341. 10.1523/JNEUROSCI.5140-13.2014 24719111 PMC6608997

[59] Beste C, Saft C. Action selection in a possible model of striatal medium spiny neuron dysfunction: behavioral and EEG data in a patient with benign hereditary chorea. Brain Struct Funct. 2015;220: 221–228. 10.1007/s00429-013-0649-9 24135770

[60] Steenbergen L, Sellaro R, Stock A-K, Verkuil B, Beste C, Colzato LS. Transcutaneous vagus nerve stimulation (tVNS) enhances response selection during action cascading processes. Eur. Neuropsychopharmacol. 10.1016/j.euroneuro.2015.03.015 25869158

[61] Verbruggen F, Schneider DW, Logan GD. How to stop and change a response: the role of goal activation in multitasking. J Exp Psychol-Hum Percept Perform. 2008;34: 1212–1228. 10.1037/0096-1523.34.5.1212 18823206

[62] Logan GD, Cowan WB. On the ability to inhibit thought and action: A theory of an act of control. Psychol Rev. 1984;91: 295. 10.1037/a0035230 24490789

[63] Logan G. D. On the ability to inhibit thought and action: A users' guide to the stop signal paradigm In: Dagenback D, Carr TH, editors. Inhibitory processes in attention, memory, and language. San Diego, CA, US: Academic Press; 1994 pp. 189–239.

[64] Kane MJ, Engle RW. The role of prefrontal cortex in working-memory capacity, executive attention, and general fluid intelligence: An individual-differences perspective. Psychon Bull Rev. 2002;9: 637–671. 12613671 10.3758/bf03196323

[65] Engle RW, Tuholski SW, Laughlin JE, Conway AR. Working memory, short-term memory, and general fluid intelligence: a latent-variable approach. J Exp Psychol Gen. 1999;128: 309 10513398 10.1037//0096-3445.128.3.309

[66] Raven JC, Court JH. Raven's progressive matrices and vocabulary scales Oxford: Psychologists Press; 1998.

[67] Pohl C, Kunde W, Ganz T, Conzelmann A, Pauli P, Kiesel A. Gaming to see: action video gaming is associated with enhanced processing of masked stimuli. Front Psychol 2014; 5. 10.3389/fpsyg.2014.00070 PMC3913992 24550879

[68] Sheehan DV, Lecrubier Y, Sheehan KH, Amorim P, Janavs J, Weiller E. et al The Mini-International Neuropsychiatric Interview (M.I.N.I.): the development and validation of a structured diagnostic psychiatric interview for DSM-IV and ICD-10. J Clin Psychiat. 1998;59: 22–23. 9881538

[69] Colzato LS, Kool W, Hommel B. Stress modulation of visuomotor binding. Neuropsychologia. 2008;46: 1542–1548. 10.1016/j.neuropsychologia.2008.01.006 18295287

[70] Colzato LS, Ruiz MJ, van den Wildenberg WPM, Hommel B. Khat use is associated with impaired working memory and cognitive flexibility. PloS one 2011;6: e20602 10.1371/journal.pone.0020602 21698275 PMC3115937

[71] Keizer AW, Verschoor M, Verment R, Hommel B. The effect of gamma enhancing neurofeedback on measures of feature-binding flexibility and intelligence. Int J Psychophysiol. 2010;75: 25–32. 10.1016/j.ijpsycho.2009.10.011 19895855

[72] Steenbergen L, Sellaro R, Stock A-K, Beste C, Colzato LS. γ-Aminobutyric acid (GABA) administration improves action selection processes: a randomised controlled trial. Sci Rep. 2015;5. 10.1038/srep12770 PMC4521208 26227783

[73] Yildiz A, Wolf OT, Beste C. Stress intensifies demands on response selection during action cascading processes. Psychoneuroendocrinology. 2014;42: 178–187. 10.1016/j.psyneuen.2014.01.022 24636514

[74] Beste C, Stok A-K, Epplen JT, Arning L. On the relevance of the NPY2-receptor variation for modes of action cascading processes. NeuroImage 2014;102: 558–564. 10.1016/j.neuroimage.2014.08.026 25157429

[75] Bejjanki VR, Zhang R, Li R, Pouget A, Green CS, Lu ZL, Bavelier D. Action video game play facilitates the development of better perceptual templates. Proc Natl Acad Sci U S A. 2014;111: 16961–16966. 10.1073/pnas.1417056111 25385590 PMC4250112

[76] Mückschel M, Stock A-K, Beste C. Different strategies, but indifferent strategy adaptation during action cascading. Sci Rep. 2015 10.1038/srep09992 PMC4649999 25950375

[77] Ferguson CJ. Video Games and Youth Violence: A Prospective Analysis in Adolescents. J Youth Adolesc. 2011;40: 377–391. 10.1007/s10964-010-9610-x 21161351

[78] Kristjánsson Á. The case for causal influences of action videogame play upon vision and attention. Atten Percept Psychophys. 2013;75: 667–672. 10.3758/s13414-013-0427-z 23386038

[79] Sherry JL, Rosaen S, Bowman ND, Huh S. Cognitive skill predicts video game ability. Paper presented at the Annual Meeting of the International Communication Association, Dresden, Germany. 2006, June.

[80] Bowman ND, Weber R, Tamborini R, Sherry JL. Facilitating game play: How others affect performance at and enjoyment of video games. J Media Psychol. 2013;16: 39–64.

[81] Sherry JL, Bowman ND. Computer games and child development In: Donsback W, Editor. International encyclopedia of communication. Oxford: Blackwell; (In press).

[82] Stock A-K, Gohil K, Beste C. Age-related differences in task goal processing strategies during action cascading. Brain Struct Funct. 2015 10.1007/s00429-015-1071-2 26025200

[83] Green CS, Strobach T, Schubert T. On methodological standards in training and transfer experiments. Psychol Res. 2014;78: 756–772. 10.1007/s00426-013-0535-3 24346424

[84] Schubert T, Strobach T. Video game experience and optimized executive control skills—On false positives and false negatives: Reply to Boot and Simons (2012). Acta Psychologica. 2012;141: 278–280.

[85] Mishra J, Zinni M, Bavelier D, Hillyard SA. Neural basis of superior performance of action videogame players in an attention-demanding task. J Neurosci. 2011;31: 992–998. 10.1523/JNEUROSCI.4834-10.2011 21248123 PMC6632922

[86] Rogers R, Bowman ND, Oliver MB. It’s not the model that doesn’t fit, it’s the controller! The role of cognitive skills in understanding the links between natural mapping, performance, and enjoyment of console video games. Comput Human Behav. 2015;49: 588–596.

[87] Chmielewski WX, Yildiz A, Beste C. The neural architecture of age-related dual-task interferences. Front Aging Neurosci 2014;6: 193 10.3389/fnagi.2014.00193 25132818 PMC4116785

[88] Verhaegen P, Borchelt M, Smith J. Relation between cardiovascular and metabolic disease and cognition in very old age: cross-sectional and longitudinal findings from the berlin aging study. Health Psychol. 2003;22:, 559 14640852 10.1037/0278-6133.22.6.559

[89] Verhaeghen P, Cerella J. Aging, executive control, and attention: a review of meta-analyses. Neurosci Biobehav Rev. 2002;26: 849–857. 12470697 10.1016/s0149-7634(02)00071-4

